# Inhibition of Mitochondrial Fission by Mdivi-1 Alleviates Doxorubicin-Induced Nephrotoxicity in a Rat Model of D-Galactose-Induced Accelerated Renal Aging

**DOI:** 10.3390/biom16081112

**Published:** 2026-07-29

**Authors:** Anongporn Kobroob, Chayodom Maneechote, Sivaporn Sivasinprasasn, Nipon Chattipakorn, Orawan Wongmekiat

**Affiliations:** 1 Division of Physiology, School of Medical Sciences, University of Phayao, Phayao 56000, Thailand; anongporn.ko@up.ac.th; 2 Cardiac Electrophysiology Research and Training Center, Faculty of Medicine, Chiang Mai University, Chiang Mai 50200, Thailand; chayodom.man@cmu.ac.th; 3 School of Medicine, Mae Fah Luang University, Chiang Rai 57100, Thailand; sivaporn.siv@mfu.ac.th; 4 Cardiac Electrophysiology Unit, Department of Physiology, Faculty of Medicine, Chiang Mai University, Chiang Mai 50200, Thailand; nipon.chat@cmu.ac.th; 5 Integrative Renal Research Unit, Department of Physiology, Faculty of Medicine, Chiang Mai University, Chiang Mai 50200, Thailand

**Keywords:** doxorubicin, nephrotoxicity, Mdivi-1, mitochondrial dynamics, renal aging, oxidative stress, inflammation, apoptosis

## Abstract

The clinical use of doxorubicin (DOX), an effective chemotherapeutic drug for breast cancer, is limited by off-target nephrotoxicity, which is exacerbated in elderly patients. While excessive mitochondrial fission is known to contribute to DOX-induced cardiotoxicity, its role in DOX-induced nephrotoxicity, particularly in the context of renal aging, remains unclear. To explore this and investigate the therapeutic potential of the mitochondrial fission inhibitor Mdivi-1, female Wistar rats with D-galactose-induced accelerated renal aging were allocated into four groups: vehicle, DOX, DOX+Mdivi-1 co-treatment, and DOX+Mdivi-1 post-treatment. DOX administration resulted in significant renal dysfunction, oxidative stress, inflammation, and histopathological damage. Mitochondrial dysfunction along with the increased expression of fission protein, decreased fusion proteins, and activation of apoptotic markers and PINK1 expression were also evident. Remarkably, both co-treatment and post-treatment with Mdivi-1 comparably and significantly attenuated DOX-induced renal damage. This study suggests that Mdivi-1 mitigates DOX-induced nephrotoxicity in the aged kidney by modulating mitochondrial dynamics, suppressing oxidative stress and inflammation, and inhibiting apoptosis. These findings highlight mitochondrial fission as a promising therapeutic target for the treatment of doxorubicin toxicity and provide further support for the possible use of Mdivi-1 as a therapeutic strategy to protect the kidneys of elderly breast cancer patients undergoing chemotherapy.

## 1. Introduction

Doxorubicin (DOX) is an anthracycline drug widely used for the treatment of several types of cancers. Specifically, it remains one of the most potent and extensively utilized chemotherapeutic agents for breast cancer [1]. However, its clinical application is limited by cumulative dose-dependent toxicity, notably cardiotoxicity and nephrotoxicity [2,3]. Although DOX-induced cardiotoxicity is widely documented, nephrotoxicity has emerged as another critical complication. Because kidneys are the primary route for DOX clearance, the drug heavily concentrates within the renal cortex. This accumulation causes severe renal structural and functional damage [4], leading to multiorgan damage that significantly impairs patient quality of life and limits therapeutic efficacy.

DOX-induced renal injury is closely associated with increased oxidative stress, inflammation, and the activation of apoptotic pathways in renal tubular epithelial cells, ultimately leading to kidney dysfunction [4]. At the cellular level, mitochondrial dysfunction is considered a central event in the pathogenesis of DOX-induced cytotoxicity, contributing to impaired bioenergetics and increased cell death [3,4,5,6,7]. Because renal tubular cells rely heavily on mitochondrial oxidative phosphorylation for reabsorption and metabolic functions, they are exceptionally vulnerable to DOX accumulation [4]. Exposure to DOX disrupts the mitochondrial electron transport chain, generating excessive reactive oxygen species (ROS) that lead to lipid peroxidation, mitochondrial DNA damage and ultimately renal cell apoptosis [3,4]. Currently, effective preventive strategies against this renal complication remain limited, highlighting the urgent need for novel protective agents.

Mitochondrial dynamics, a delicate balance between fission and fusion processes, are critical for maintaining mitochondrial integrity and cellular homeostasis [8]. Dysregulated mitochondrial fission has been implicated in chemotherapy-induced organ toxicity, including doxorubicin-induced cardiotoxicity, where excessive fission promotes apoptosis [8,9]. Selective inhibition of the master mitochondrial fission protein dynamin-related protein 1 (DRP1) by compounds such as Mdivi-1 has shown protective effects by attenuating mitochondrial fragmentation and cell death [10,11,12]. Despite growing evidence of mitochondrial fission’s role in chemotherapy toxicity, its contribution to DOX-induced nephrotoxicity remains poorly understood.

Aging is a major risk factor for the development and progression of kidney disease, characterized by a gradual decline in functional renal mass accompanied by structural and functional alterations, including glomerulosclerosis, tubular atrophy, interstitial fibrosis, increased oxidative stress, and mitochondrial dysfunction [13,14]. Aged kidneys are particularly susceptible to secondary insults, which may accelerate renal injury and impair recovery capacity [13,14]. Given that the global population of aging women is projected to exceed one billion by 2050, surpassing men in the same age groups [15], and that breast cancer is the leading cause of cancer-related death among women worldwide [1], it is critical to investigate therapeutic strategies tailored to this demographic.

The D-galactose-induced accelerated aging model replicates key features of natural aging and has been widely used to study age-related pathologies [14,16]. To investigate the compounding effects of DOX-induced nephrotoxicity under conditions of renal aging without relying on chronological age, a female rat model of D-galactose-induced accelerated renal aging was used in this study. This model effectively mimics the age-related susceptibility and increased vulnerability of renal tissues to toxic insults [14,16]. Furthermore, while most protective strategies focus on early intervention, clinical reality often requires treatment after injury has already commenced. To address clinical feasibility, we evaluated the therapeutic potential of Mdivi-1 through both co-treatment (coTx) and post-treatment (postTx) regimens, focusing on its capacity to modulate mitochondrial dynamics, mitophagy, mitochondrial function, and apoptosis. This research aims to provide novel insights into mitochondrial-targeted therapies that may mitigate chemotherapy-induced renal injury in elderly female cancer patients, thereby improving clinical outcomes and quality of life.

## 2. Materials and Methods

### 2.1. Chemicals, Reagents, and Antibodies

Doxorubicin hydrochloride injection (DOX; Product No. 100161) was obtained from Fresenius Kabi Oncology Ltd. (Haryana, India). Mitochondrial Division Inhibitor 1 (Mdivi-1; Cat. No. HY-15886) was bought from MedChemExpress LLC (Monmouth Junction, NJ, USA). D-(+)-Galactose (D-gal; Cat. No. G6404), and all other analytical-grade chemicals and reagents (unless otherwise specified) were procured from Sigma-Aldrich^®^ (Merck KGaA, Darmstadt, Germany).

Antibodies against B-cell lymphoma 2 (Bcl-2; Cat. No. ab196495), mitofusin 1 (Mfn1; Cat. No. ab221661) and PTEN-induced putative kinase 1 (PINK1; Cat. No. ab326097) were acquired from Abcam (Cambridge, MA, USA).

Antibodies against optic atrophy 1 (OPA1; Cat. No. 80471), dynamin-related protein 1 (DRP1; Cat. No. 5391), phospho-DRP1 at Ser616 (pDRP1Ser616; Cat. No. 63940), mitofusin 2 (Mfn2; Cat. No. 9482), Bcl-2-associated X protein (Bax; Cat. No. 2772), voltage-dependent anion channel (VDAC; Cat. No. 4661), β-actin (Cat. No. 8457), and both procaspase-3 and cleaved caspase-3 (Cat. No. 14220) were purchased from Cell Signaling Technology (Danvers, MA, USA).

### 2.2. Animals

Female Wistar rats (300–350 g) were obtained from the Nomura Siam International, Bangkok, Thailand, and housed in the Laboratory Animal Center at Chiang Mai University under standard temperature and humidity conditions with a 12 h light–dark cycle. Food and water were given *ad libitum*. The animals were allowed one week to acclimatize before starting the experiment. All animal procedures and experimental protocols were reviewed and approved by the Laboratory Animal Center of Chiang Mai University (Permit No. 2565/RT-0010) and were conducted in accordance with the guidelines outlined by the National Institutes of Health (NIH) for the care and use of laboratory animals.

### 2.3. Experimental Designs

#### 2.3.1. Experiment 1: Validation of D-Galactose-Induced Accelerated Renal Aging-like Phenotype

To confirm the establishment of an accelerated renal aging-like phenotype, rats were divided into two groups (*n* = 6 each): vehicle control (Veh-CON) and D-galactose control (D-gal-CON). The D-gal-CON group received subcutaneous injections of D-galactose (150 mg/kg/day) for 4 weeks [14,17], while the Veh-CON group received an equivalent volume of 0.9% normal saline. Renal function, oxidative stress, mitochondrial function, and histopathological changes were assessed after the treatment period.

#### 2.3.2. Experiment 2: Doxorubicin-Induced Nephrotoxicity and Mdivi-1 Treatment Protocol

This experiment was conducted after successful validation of the D-galactose-induced accelerated renal aging model. A total of 32 rats were included. All rats received D-galactose (150 mg/kg/day, subcutaneously) for 4 consecutive weeks and were then randomly assigned to four groups (*n* = 8 each).

Vehicle control group (Veh): Rats received intraperitoneal (i.p.) injections of 0.9% normal saline on days 0, 4, 8, 15, 22, and 29, followed by a 4-week observation period.

Doxorubicin group (DOX): Rats received doxorubicin (3 mg/kg, i.p.) on the same schedule as the Veh group, followed by a 4-week observation period. The dose and regimen of doxorubicin were chosen based on a previous report showing that it effectively and safely induced cardiotoxicity with no mortality [11].

Doxorubicin+Mdivi-1 coTx group (DOX+Mdivi-1 coTx): Rats received doxorubicin as above, together with Mdivi-1 (1.2 mg/kg/day, i.p.) administered concurrently during the 4-week DOX treatment period. The dose of Mdivi-1 was selected according to previous publication showing that it effectively protects against DOX-induced myocardial damage [11].

Doxorubicin+Mdivi-1 postTx group (DOX+Mdivi-1 postTx): Rats received doxorubicin as above, followed by Mdivi-1 (1.2 mg/kg/day, i.p.) administered for 4 weeks after completion of DOX treatment. 

At the end of the experiment, blood samples were collected from abdominal aorta under thiopental anesthesia (80 mg/kg, i.p.). Serum was separated by centrifugation (1500× *g* for 10 min at 4 °C) and stored at −80 °C until biochemical analysis. Both kidneys were perfused with ice-cold phosphate-buffered saline (pH 7.4) and quickly harvested. One kidney was taken immediately for studying mitochondrial function. A portion of kidney was fixed in 10% neutral buffered formalin for light microscopy, another portion was fixed in 2.5% glutaraldehyde in 0.1 M phosphate buffer (pH 7.4) at 4 °C overnight for electron microscopy. The remaining kidney tissues were snap-frozen in liquid nitrogen and stored at −80 °C for further analyses.

### 2.4. Biochemical Analyses

#### 2.4.1. Assessments of Renal Functions and Tubular Injury Biomarker

Renal function was assessed by measuring blood urea nitrogen (BUN) and serum creatinine using an automatic analyzer (Beckman Coulter, Inc., Brea, CA, USA). Kidney injury molecule-1 (KIM-1), a marker of tubular injury, was detected by a sandwich ELISA kit (Cat. No. ab119597, Abcam, Waltham, MA, USA), as per the manufacturer’s instruction.

#### 2.4.2. Assessments of Renal Oxidative Stress

Levels of malondialdehyde (MDA), a lipid peroxidation product, and glutathione (GSH), a non-enzymatic antioxidant, in kidney tissue were used to determine renal oxidative stress. MDA was measured using the TBARS kit (Cat. No. 10009055, Cayman Chemical Company, Ann Arbor, MI, USA), and GSH was measured using the QuantiChrom™ Glutathione Assay Kit (Cat. No. DIGT-250, Bioassay Systems, Hayward, CA, USA), according to the manufacturer’s instructions.

#### 2.4.3. Assessments of Renal Inflammation

The kidney tissue levels of tumor necrosis factor-alpha (TNF-α) and interleukin-1beta (IL-1β) were assayed using Rat TNF-alpha (Cat. No. ab255730) and IL-1 beta (Cat. No. ab236712) ELISA kits (Abcam, Waltham, MA, USA), respectively, according to the manufacturer’s instructions.

### 2.5. Mitochondrial Studies

#### 2.5.1. Isolation of Mitochondrial Fractions and Protein Quantification

Renal cortical tissues were homogenized in ice-cold lysis buffer (230 mM mannitol, 70 mM sucrose, 1 mM EDTA, and 10 mM Tris-HCl, pH 7.4), and mitochondria were isolated by differential centrifugation as previously described [18]. The isolated mitochondrial pellet was resuspended in respiration buffer (250 mM sucrose, 5 mM KH_2_PO_4_, 10 mM Tris-HCl, and 2 mg/mL BSA, pH 7.2). Mitochondrial protein concentration was quantified using the bicinchoninic acid (BCA) assay with BSA as the calibration standard.

#### 2.5.2. Determination of Mitochondrial Reactive Oxygen Species (ROS)

Mitochondrial ROS production was determined using the fluorogenic probe 2′,7′-dichlorofluorescein diacetate (DCFDA) as previously described [18]. Isolated mitochondria were incubated with 2 μM DCFDA at 25 °C for 60 min, and DCF fluorescence, the oxidized product of DCFDA, was measured using a microplate reader (excitation 485 nm, emission 530 nm). ROS levels were expressed as arbitrary fluorescence units.

#### 2.5.3. Determination of Mitochondrial Membrane Potential (MMP)

Mitochondrial membrane potential was assessed using the lipophilic cationic fluorescent dye 5,5′,6,6′-tetrachloro−1,1′,3,3′-tetraethylbenzimidazolylcarbocyanine iodide (JC-1) as previously described [18]. Briefly, isolated mitochondria were incubated with 310 nM JC-1 at 37 °C for 30 min. Fluorescence was measured using a microplate reader to detect the red fluorescence emitted by JC-1 aggregates (excitation 485 nm, emission 590 nm), which accumulate in polarized mitochondria, and the green fluorescence emitted by JC-1 monomers (excitation 485 nm, emission 530 nm), which predominate in depolarized mitochondria. MMP was expressed as the ratio of red to green fluorescence intensity (JC-1 aggregates/monomers), with a decrease in the red/green fluorescence ratio indicating mitochondrial membrane depolarization.

#### 2.5.4. Determination of Mitochondrial Swelling

Mitochondrial swelling was assessed by monitoring changes in absorbance at 540 nm, as previously described [18]. Briefly, isolated mitochondria were incubated at 25 °C, and absorbance was measured every minute for 15 min using a microplate reader (Synergy™ H4, BioTek^®^ Instruments, Winooski, VT, USA). Mitochondrial swelling was quantified as a decrease in absorbance at 540 nm, reflecting increased mitochondrial volume and reduced light scattering.

### 2.6. Western Blot Analysis

Renal cortical tissues were homogenized in extraction buffer containing 20 mM Tris (pH 6.8), 1 mM sodium orthovanadate, 5 mM sodium fluoride, and protease inhibitors. Protein concentration was determined using a Bio-Rad protein assay kit (Bio-Rad Laboratories, Hercules, CA, USA). Equal amounts of protein (30 µg) were denatured, separated by 10% SDS-PAGE, and transferred onto PVDF membranes using a wet-transfer system. Membranes were blocked with 5% bovine serum albumin in TBST and incubated overnight at 4 °C with primary antibodies against DRP1, pDRP1^Ser616^, Mfn1, Mfn2, OPA1, PINK1, Bax, Bcl-2, procaspase-3, cleaved caspase-3, β-actin, and VDAC. β-actin was used as the loading control for cytosolic and apoptosis-related proteins, whereas VDAC served as the loading control for mitochondrial-associated proteins. After incubation with appropriate horseradish peroxidase-conjugated secondary antibodies for 1 h at room temperature, immunoreactive bands were visualized using enhanced chemiluminescence (ECL). Images were acquired using a ChemiDoc Touch Imaging System, and band intensities were quantified using ImageJ software version 1.54d (National Institutes of Health, Bethesda, MD, USA).

### 2.7. Histopathological Studies

#### 2.7.1. Light Microscopic Studies

Kidney tissues were fixed in 10% neutral buffered formalin, dehydrated through a graded ethanol series, cleared in xylene, and embedded in paraffin wax. Serial sections (4 μm) were deparaffinized, rehydrated, and stained with hematoxylin and eosin (H&E) and Masson’s trichrome for light microscopic examination.

To perform semi-quantitative histopathological analysis, tubular injury was evaluated on H&E-stained renal sections by a pathologist blinded to the experimental groups. Kidney injury was defined by the presence of tubular dilatation, luminal obstruction, cast formation, tubular cell apoptosis and/or necrosis, and inflammatory cell infiltration. The severity of structural injury in each microscopic field was graded using a semi-quantitative scoring system based on the percentage of the affected tubular area, adapted with slight modifications [19]. Individual fields were assigned a score from 0 to 4 as follows: score 0 = no change or affected area < 10%; score 1 = affected area 10–25%; score 2 = affected area 26–50%; score 3 = affected area 51–75%; and score 4 = affected area > 75%. For each animal, 10 randomly selected non-overlapping cortical fields under high-power magnification were evaluated, and the individual scores were summed to yield a cumulative arbitrary tubular injury score (maximum possible score of 40).

#### 2.7.2. Electron Microscopic Studies

The electron microscopic procedure was performed as previously described [18]. Briefly, glutaraldehyde-fixed kidney tissues were rinsed in cacodylate buffer and post-fixed in 2% osmium tetroxide for 1 h at room temperature. Samples were then dehydrated through a graded ethanol series, cleared in propylene oxide, and embedded in Epon resin using an EMbed-812 embedding kit (Electron Microscopy Sciences, Hatfield, PA, USA). Ultrathin sections (60–80 nm) were cut, mounted on copper grids, and stained with uranyl acetate followed by lead citrate. Sections were examined using a JEM-2200FS transmission electron microscope (JEOL, Tokyo, Japan).

Transmission electron microscopy (TEM) images of renal proximal tubular epithelial cells were quantitatively analyzed using Aperio ImageScope software (Version 12.3.2, Leica Biosystems, Wetzlar, Germany) as previously described with modifications [20,21]. Five randomly selected, non-overlapping TEM fields per animal (8 animals/group) were analyzed at an identical magnification (×15,000) by an investigator blinded to the experimental groups. All identifiable mitochondrial profiles within the selected fields were included for analysis. Mitochondrial profiles with sufficiently preserved boundaries were manually outlined for morphometric assessment, including major axis length, minor axis width, and aspect ratio (major axis/minor axis). Mitochondria were classified as morphologically altered when they exhibited one or more ultrastructural abnormalities, such as swelling, irregular morphology, disrupted or reduced cristae, an electron–lucent matrix, vacuolization, or mitochondrial membrane disruption. The percentage of altered mitochondria per animal was calculated as follows: (number of morphologically altered mitochondria/total number of identifiable mitochondria analyzed) × 100. For statistical analysis, measurements from mitochondria within each animal were averaged, and each animal was considered the experimental unit.

### 2.8. Statistical Analysis

Data are presented as mean ± SEM. The normality of data distribution and homogeneity of variance were verified using the Shapiro–Wilk and Levene’s tests, respectively. Differences among groups were analyzed using one-way analysis of variance (ANOVA) followed by Tukey’s *post hoc* test for multiple comparisons. A *p*-value < 0.05 was considered statistically significant. Analyses were conducted using SPSS version 25 (IBM Corporation, Armonk, NY, USA).

## 3. Results

### 3.1. Experiment 1: Validation of D-Galactose-Induced Accelerated Renal Aging-like Phenotype

#### D-Galactose Successfully Induced Accelerated Renal Aging-like Phenotype

**Figure 1:**
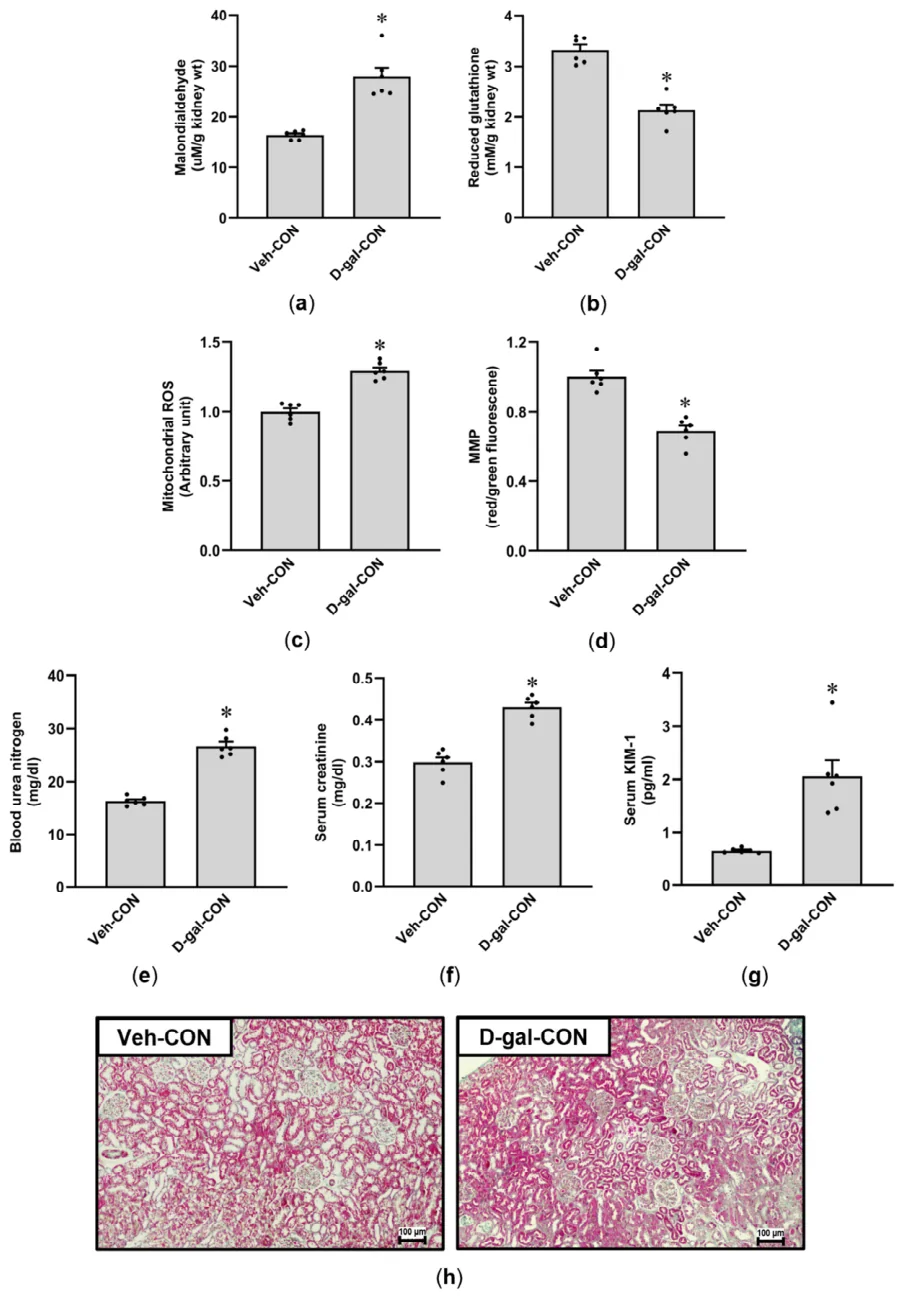
Effects of D-galactose (D-gal) administration on oxidative stress, mitochondrial function, renal function, and histomorphological alterations. (**a**) Malondialdehyde (MDA); (**b**) reduced glutathione (GSH); (**c**) mitochondrial ROS production; (**d**) mitochondrial membrane potential (MMP); (**e**) blood urea nitrogen (BUN); (**f**) serum creatinine; (**g**) serum kidney injury molecule-1 (KIM-1); and (**h**) Masson’s trichrome staining (scale bar 100 µm). Values are means ± SEM (*n* = 6 each). Veh-CON: healthy rats received vehicle; D-gal-CON: healthy rats received D-galactose. * *p* < 0.05 vs. Veh-CON.

Rats given D-gal had higher kidney tissue levels of MDA as well as lower GSH, higher mitochondrial ROS generation, and lower MMP (all *p* < 0.05) when compared to the healthy control rats (Figure 1a–d), suggesting that D-gal caused oxidative stress in the kidney. These results were consistent with a significant decline in renal function (increased BUN, serum creatinine, and KIM-1, Figure 1e–g) and the existence of fibrosis in Masson’s trichrome-stained kidney segment of D-gal-CON rats compared to Veh-CON rats (Figure 1h). All evidence suggests the successful development of a model of D-gal-induced accelerated renal aging-like phenotype in our study.

### 3.2. Experiment 2: Doxorubicin-Induced Nephrotoxicity and Mdivi-1 Treatment Protocol

#### 3.2.1. Mdivi-1 Mitigates DOX-Induced Renal Dysfunction

**Figure 2:**
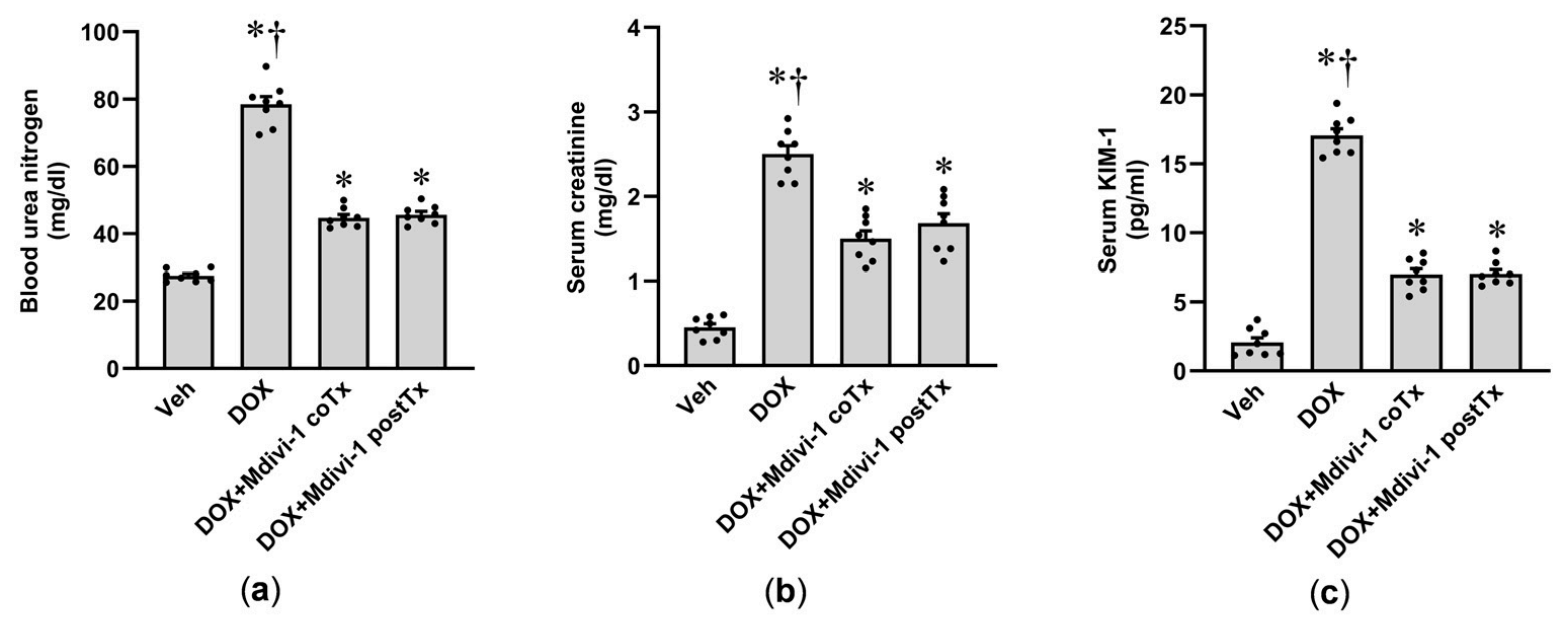
Effects of doxorubicin (DOX) and Mdivi-1 treatment on renal function in D-galactose-induced accelerated renal aging rats. (**a**) Blood urea nitrogen (BUN); (**b**) serum creatinine; and (**c**) kidney injury molecules-1 (KIM-1). Values are means ± SEM (*n* = 8 each). Veh: D-gal rats received vehicle; DOX: D-gal rats received DOX; DOX+Mdivi-1 coTx; D-gal rats received DOX and coTx with Mdivi-1; DOX+Mdivi-1 postTx: D-gal rats received DOX and postTx with Mdivi-1, respectively. * *p* < 0.05 vs. Veh, † *p* < 0.05 vs. DOX+Mdivi-1 coTx and postTx, respectively.

Figure 2 demonstrates the effects of DOX and Mdivi-1 treatment on renal function of all D-galactose-induced accelerated renal aging rats. As expected, all the groups that received DOX had significantly greater serum levels of BUN, creatinine, and KIM-1 (all *p*  <  0.05) than the D-galactose-Veh control group (Figure 2a–c, respectively). In the Mdivi-1 treated groups, regardless of whether they received coTx or postTx, the levels of these renal functional indexes were very similar, being markedly and significantly lower than those observed in the group that received DOX alone (all *p*  <  0.05).

#### 3.2.2. Mdivi-1 Attenuates DOX-Induced Kidney Histomorphological Damages

**Figure 3:**
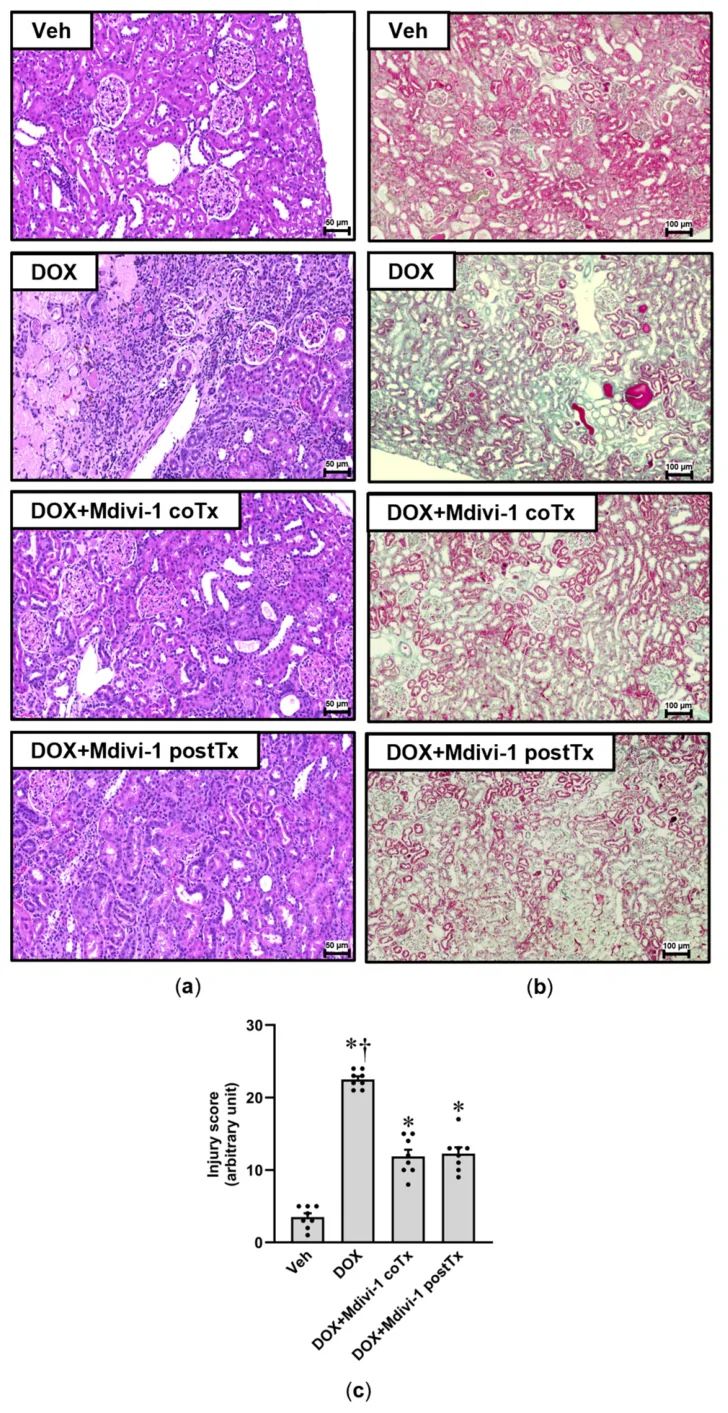
Effects of doxorubicin (DOX) and Mdivi-1 treatment on kidney histopathological changes in D-galactose-induced accelerated renal aging rats. (**a**) Hematoxylin–Eosin (H&E, scale bar 50 µm) staining; (**b**) Masson’s trichrome staining (scale bar 100 µm); and (**c**) kidney injury score. Values are means ± SEM (*n* = 8 each). Veh: D-gal rats received vehicle; DOX: Dgal-rats received DOX; DOX+Mdivi-1 coTx; D-gal rats received DOX and coTx with Mdivi-1; DOX+Mdivi-1 postTx: D-gal rats received DOX and postTx with Mdivi-1, respectively. * *p* < 0.05 vs. Veh, † *p* < 0.05 vs. DOX+Mdivi-1 coTx and postTx, respectively.

H&E and Masson’s trichrome-stained renal cortical tissues from each of the groups under investigation are displayed in Figure 3a,b, respectively. In the D-gal vehicle control group, H&E-stained kidney section shows relatively preserved renal architecture with intact glomeruli and tubules, though there is mild tubular dilation or early cell swelling, typical of D-gal-induced accelerated renal aging. Masson’s trichrome also showed minor, baseline blue collagen staining confined primarily to the perivascular and peritubular regions. This confirms that D-gal causes low-grade, chronic changes rather than massive structural destruction. In contrast, the kidney section of the DOX alone group exhibited a wide variety of significant abnormalities that were consistent with their renal functional alterations. Tubular dilatation and atrophy in some areas, tubular obstruction, hyaline cast formation, apoptosis and/or necrosis, and inflammatory cell infiltration were observed from H&E staining. Masson’s trichrome staining also displayed an extreme accumulation of coarse blue collagen fibers throughout the interstitial spaces, highlighting advanced renal fibrosis secondary to severe tubular collapse and necrosis. These alterations were diminished when Mdivi-1 was given, either concurrently or after DOX administration. This observation was further supported by a semi-quantitative examination of the renal tubular damages (Figure 3c), which showed significantly lower injury scores in both DOX+Mdivi-1-treated groups than that of the untreated DOX group (all *p* < 0.05).

#### 3.2.3. Mdivi-1 Alleviates DOX-Induced Renal Oxidative Stress and Inflammation

**Figure 4:**
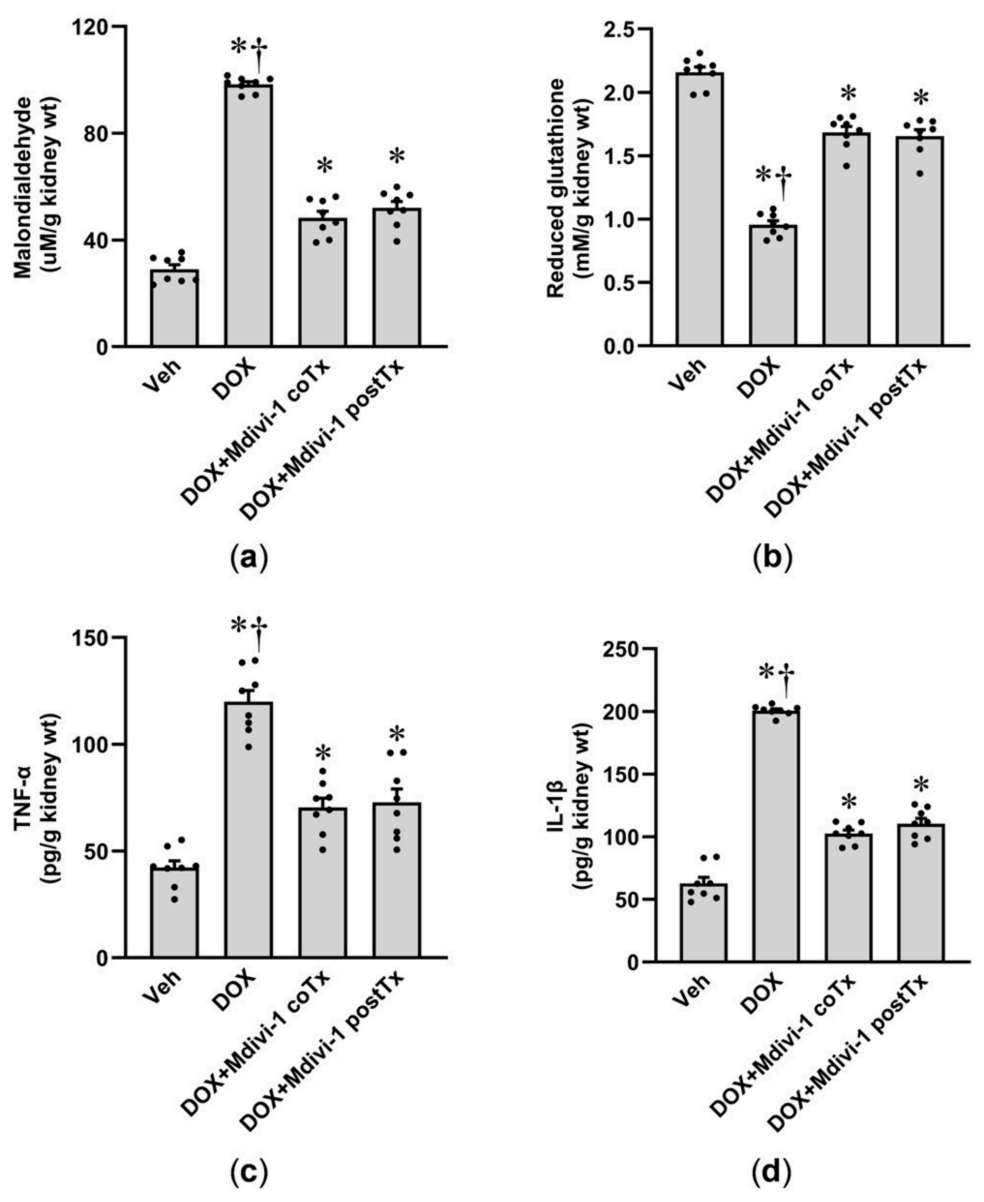
Effects of doxorubicin (DOX) and Mdivi-1 treatment on renal oxidative stress and inflammation in D-galactose-induced accelerated renal aging rats. (**a**) Malondialdehyde (MDA); (**b**) reduced glutathione (GSH); (**c**) tumor necrosis-alpha (TNF-α); and (**d**) interleukin-1beta (IL-1β). Values are means ± SEM (*n* = 8 each). Veh: D-gal rats received vehicle; DOX: D-gal rats received DOX; DOX+Mdivi-1 coTx; D-gal rats received DOX and coTx with Mdivi-1; DOX+Mdivi-1 postTx: D-gal rats received DOX and postTx with Mdivi-1, respectively. * *p* < 0.05 vs. Veh, † *p* < 0.05 vs. DOX+Mdivi-1 coTx and postTx, respectively.

The levels of a lipid peroxidation product, MDA, and a non-enzymatic antioxidant, GSH, in kidney tissue were measured to ascertain the impact of DOX and Mdivi-1 on renal oxidative stress in D-galactose-induced accelerated renal aging rats (Figure 4). All the rats exposed to DOX exhibited significantly higher kidney tissue levels of MDA (Figure 4a) but lower GSH (Figure 4b) than the rats in the D-galactose vehicle control group (all *p*  <  0.05). Regardless of the timing of treatment, Mdivi-1 given to DOX-exposed rats significantly (all *p*  <  0.05) alleviated these alterations.

Consistent with renal oxidative stress, the D-gal rats receiving DOX showed a marked rise (both *p* < 0.05) in kidney tissue levels of TNF-α and IL-1β (Figure 4c,d) compared to the D-gal vehicle rats. Both TNF-α and IL-1β levels were reduced significantly with Mdivi-1 intervention, irrespective of the time given.

#### 3.2.4. Mdivi-1 Ameliorates DOX-Induced Mitochondrial Damages

**Figure 5:**
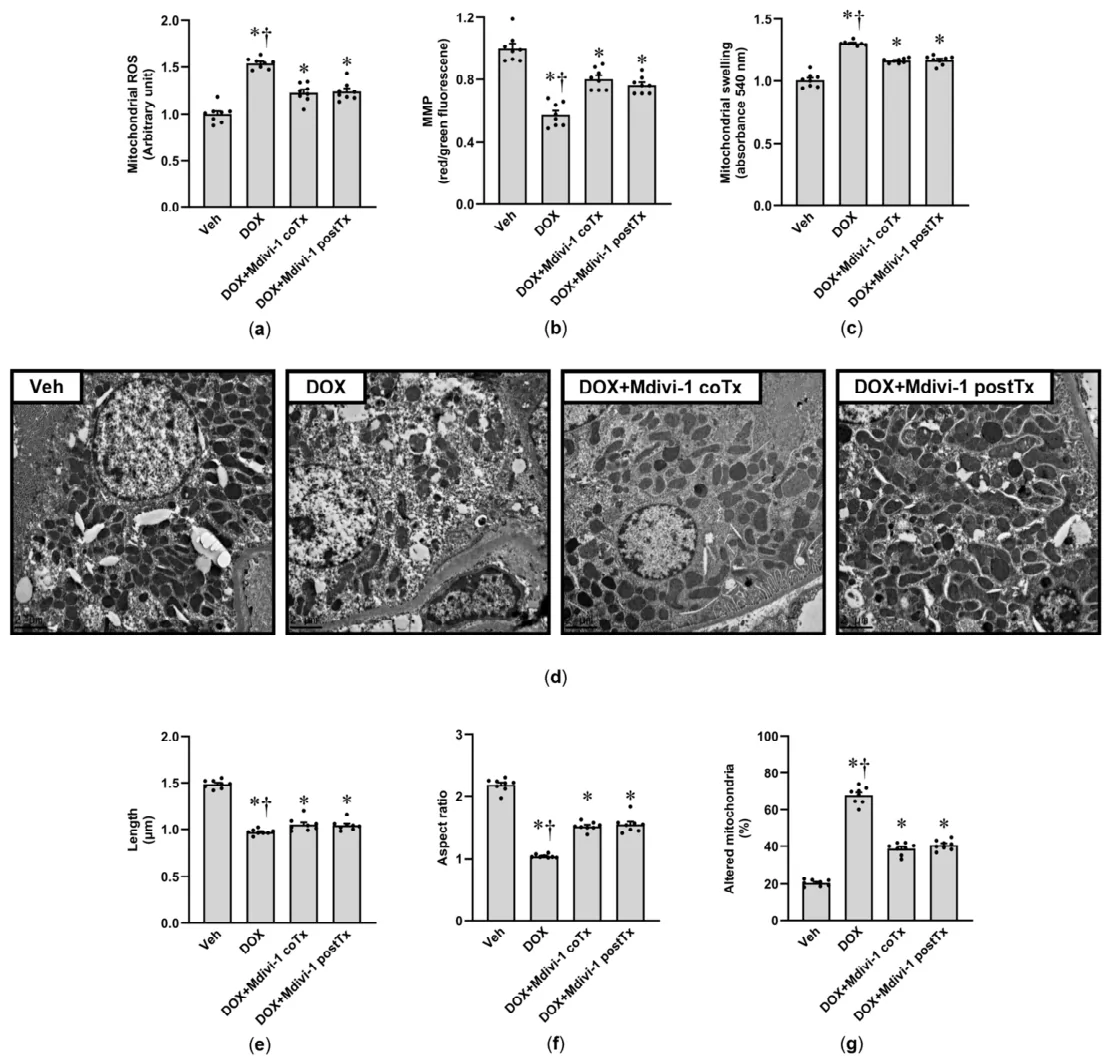
Effects of doxorubicin (DOX) and Mdivi-1 treatment on mitochondrial function and ultrastructure in D-galactose-induced accelerated renal aging rats. (**a**) Mitochondrial ROS production; (**b**) mitochondrial membrane potential (MMP); (**c**) mitochondrial swelling; (**d**) transmission electron microscopic images of proximal tubules (original magnification: 15,000×); and quantitative analysis of (**e**) mitochondrial length; (**f**) aspect ratio; and (**g**) percentage of altered mitochondria. Values are means ± SEM (*n* = 8 each). Veh: D-gal rats received vehicle; DOX: D-gal rats received DOX; DOX+Mdivi-1 coTx; D-gal rats received DOX and coTx with Mdivi-1; DOX+Mdivi-1 postTx: Dgal-rats received DOX and postTx with Mdivi-1, respectively. * *p* < 0.05 vs. Veh, † *p* < 0.05 vs. DOX+Mdivi-1 coTx and postTx, respectively.

Figure 5 shows the effects of DOX and Mdivi-1 treatments on mitochondrial function and ultrastructure of D-galactose-induced accelerated renal aging rats. A significant rise in mitochondrial ROS production (Figure 5a), a marked reduction in mitochondrial membrane potential (Figure 5b), and evidence of mitochondrial swelling (Figure 5c) were detected in the group receiving DOX alone in comparison with the group receiving vehicle only (all *p* < 0.05). These changes caused by DOX were significantly ameliorated by Mdivi-1 at an equal efficiency whether given as coTx or postTx.

In line with mitochondrial function, electron microscopic images of the D-galactose-induced accelerated renal aging rats that received DOX alone showed substantial changes in the mitochondrial ultrastructure (Figure 5d). Mitochondrial swelling with less dense cristae, fragmentation, and a considerable reduction in number were all detectable in the DOX-alone group. Administration of Mdivi-1 to the DOX-exposed rats as coTx or postTx attenuated these changes. Mitochondria appeared more elongated in shape with dense cristae, and an increase in number was observed. Quantitative TEM analysis (Figure 5e–g) confirmed these morphological changes, demonstrating a significant decrease in mitochondrial length and aspect ratio, along with an increased percentage of altered mitochondria in the DOX-alone group compared to the Veh group. Treatment with Mdivi-1 as coTx or postTx significantly mitigated all DOX-induced alterations.

#### 3.2.5. Mdivi-1 Impedes DOX-Activated Mitochondrial Dynamic Imbalance

**Figure 6:**
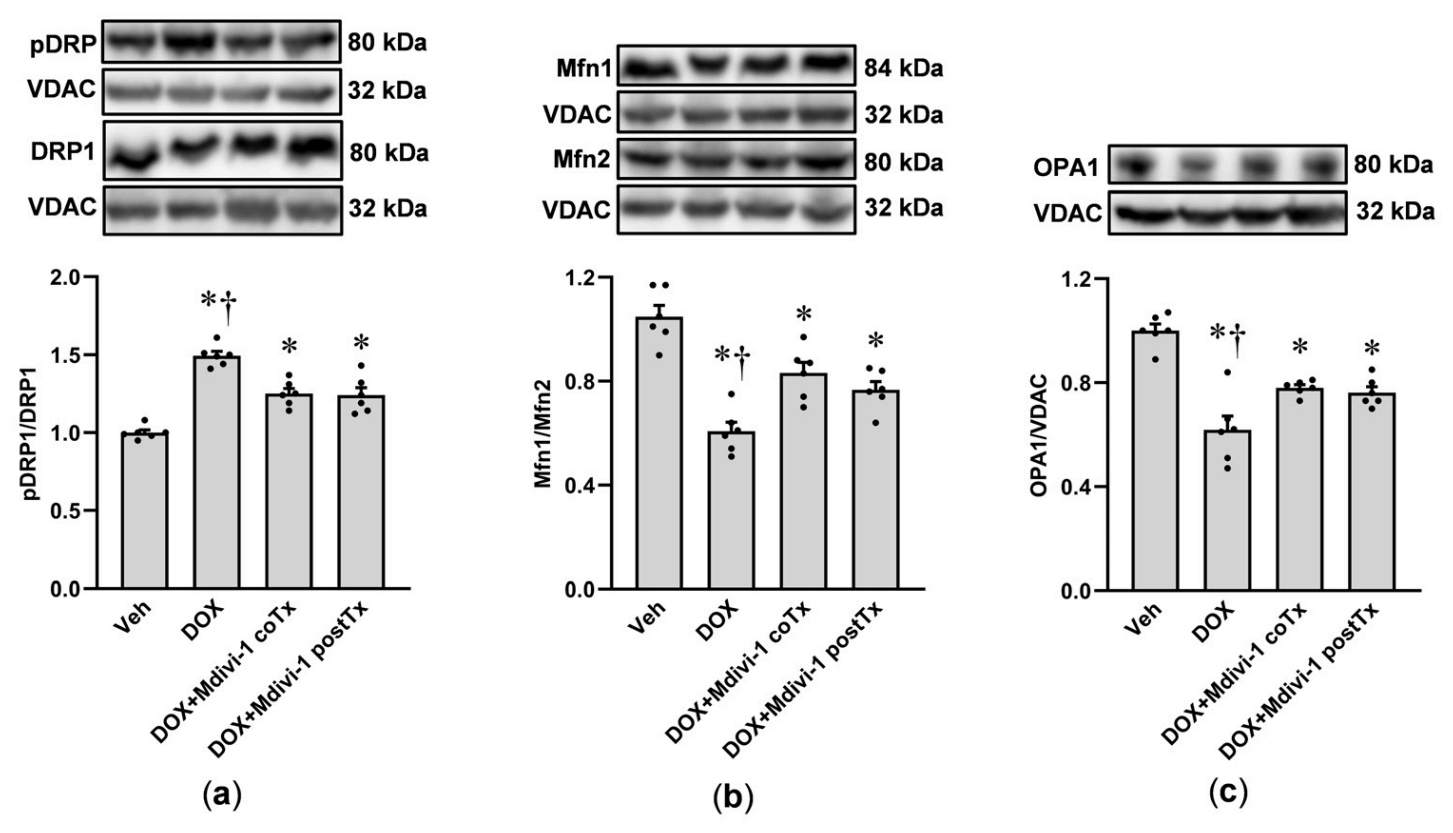
Effects of doxorubicin (DOX) and Mdivi-1 treatment on renal cortical expressions of protein involved in mitochondrial dynamic balance. (**a**) pDRP1/DRP1; (**b**) Mfn1/Mfn2; and (**c**) OPA1. VDAC was used as a loading control. The original WB images are shown in Figures S1–S3, respectively. Values are means ± SEM (*n* = 6 each). Veh: D-gal rats received vehicle; DOX: D-gal rats received DOX; DOX+Mdivi-1 coTx; D-gal rats received DOX and coTx with Mdivi-1; DOX+Mdivi-1 postTx: D-gal rats received DOX and postTx with Mdivi-1. * *p* < 0.05 vs. Veh, † *p* < 0.05 vs. DOX+Mdivi-1 coTx and postTx, respectively.

The renal cortical expressions of proteins related to mitochondrial dynamic control were evaluated, and the results are demonstrated in Figure 6. Western blot analysis revealed a significant increase in the expressions of mitochondrial fission proteins (indicated by a significant increase in the pDRP1/DRP1 ratio), while decreases in the expressions of mitochondrial fusion proteins (OPA1 and Mfn1/Mfn2 ratio) were observed in the DOX group compared to those in the Veh group (all *p* < 0.05). The imbalance of mitochondrial dynamic control caused by DOX was partially, but significantly (all *p* < 0.05), impeded by Mdivi-1 treatment. Both coTx and postTx showed a comparable degree of protection.

#### 3.2.6. Mdivi-1 Protects Against DOX-Induced Cell Death

**Figure 7:**
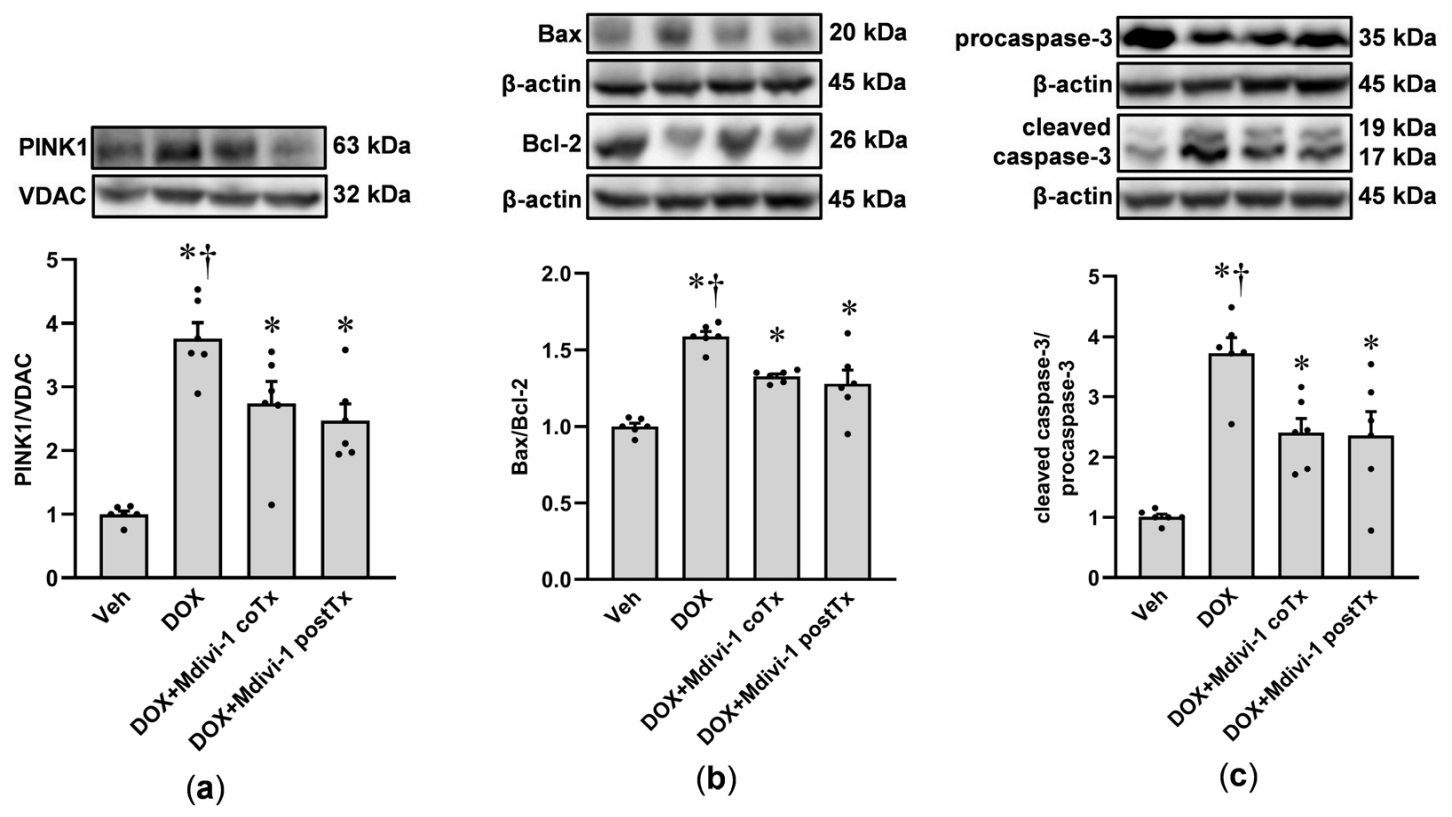
Effects of doxorubicin (DOX) and Mdivi-1 treatment on renal cortical expressions of protein involved in renal cell death pathways. (**a**) PINK1; (**b**) Bax/Bcl-2; and (**c**) cleaved caspase-3/procaspase-3. VDAC or β-actin was used as a loading control (as appropriate). The original WB images are shown in Figures S4–S6, respectively. Values are means ± SEM (*n* = 6 each). Veh: D-gal rats received vehicle; DOX: D-gal rats received DOX; DOX+Mdivi-1 coTx; D-gal rats received DOX and coTx with Mdivi-1; DOX+Mdivi-1 postTx: D-gal rats received DOX and postTx with Mdivi-1. * *p* < 0.05 vs. Veh, † *p* < 0.05 vs. DOX+Mdivi-1 coTx and postTx, respectively.

Figure 7 shows the expression of proteins linked to renal cell death pathways. The expression of the mitophagy protein PINK1 was significantly higher in all the groups that received DOX than in the Veh control group (all *p* < 0.05). DOX also had an impact on the expression of several apoptosis-related proteins. It reduced the expression of the anti-apoptotic protein Bcl-2 while increasing the expression of the pro-apoptotic proteins Bax and caspase-3 (all *p* < 0.05). Treatment with Mdivi-1, however, was able to significantly (all *p* < 0.05) mitigate these effects of DOX. The advantage of Mdivi-1 was evident regardless of when it was delivered.

## 4. Discussion

This study demonstrates that doxorubicin (DOX) induces significant nephrotoxicity in a D-galactose-induced accelerated renal aging female rat model via mechanisms involving oxidative stress, inflammation, mitochondrial dysfunction, dysregulated mitochondrial dynamics, and apoptosis. Importantly, pharmacological inhibition of mitochondrial fission using Mdivi-1 significantly ameliorated these pathological alterations with comparable efficacy between both coTx and postTx strategies. These findings support the central role of mitochondrial dynamics in DOX-induced kidney injury and highlight the inhibition of mitochondrial fission as a promising therapeutic approach.

Doxorubicin is a potent and widely used chemotherapeutic agent, though it is known to cause off-target toxicity in various organs [1,2,3]. The nephrotoxic effect of DOX is well-documented, particularly in susceptible populations such as the elderly [22,23]. In our D-galactose-induced accelerated renal aging rat model, DOX administration resulted in significant renal dysfunction, as indicated by increased BUN, serum creatinine, and the tubular injury marker KIM-1. Histopathological findings provided support to the renal functional impairment, as revealed by widespread tubular injury, including tubular dilation, atrophy, obstruction, hyaline cast formation, inflammatory cell infiltration, interstitial fibrosis, and apoptosis. Moreover, these pathological changes were closely associated with a marked increase in the pro-inflammatory cytokines TNF-α and IL-1β in renal tissues. These findings align with previous studies reporting that both glomerular and tubular damage, as well as robust inflammatory responses, are involved in DOX-induced renal injury [24,25,26].

In this study, we found that alterations in renal function after DOX administration were accompanied by an increase in MDA, a lipid peroxidation product, and a decrease in antioxidant GSH levels in renal tissue. The results indicate the involvement of oxidative stress and further validate the redox-cycling properties of DOX. Doxorubicin has been shown to localize within mitochondria where it undergoes redox cycling, generating reactive oxygen species (ROS) that damage lipids, proteins, and DNA [3,4,5,6,7,26]. Oxidative stress is both a cause and a consequence of mitochondrial dysfunction. It exacerbates direct cellular injury and contributes to the progression of tubular injury and the initiation of inflammatory and apoptotic signaling cascades. Our findings of increased TNF-α and IL-1β in the kidney tissues confirmed the development of renal inflammation following DOX administration. This was further supported by earlier reports showing that DOX exposure triggers oxidative stress and activates the NOD-like receptor family pyrin domain-containing 3 (NLRP3) inflammasome, leading to the release of pro-inflammatory cytokines such as IL-1β. DOX also activates the nuclear factor-kappa B (NF-κB) pathway by promoting the degradation of IκB proteins, thereby enhancing the transcription of inflammatory mediators such as TNF-α [5].

Mitochondria are central regulators of renal cell fate, particularly under oxidative stress conditions. In this study, we observed marked mitochondrial impairment following DOX exposure, including elevated mitochondrial ROS, decreased mitochondrial membrane potential (MMP), and mitochondrial swelling. Ultrastructural evaluation via transmission electron microscopy (TEM) confirmed these changes, revealing swollen mitochondria with structural disintegration of cristae, and a notable reduction in mitochondrial number. Quantitative TEM analysis confirmed these morphological changes, demonstrating a significant decrease in both mitochondrial length and aspect ratio, along with a higher percentage of altered mitochondria following DOX exposure. These features reflect mitochondrial damage that is strongly linked to energy depletion and the activation of apoptotic pathways. Concurrently, the observed upregulation of PINK1 suggests the activation of upstream mitophagic signaling, likely reflecting an initial compensatory response to target damaged mitochondria for degradation [27]. Collectively, our findings indicate that both oxidative stress and mitochondrial dysfunction play pivotal roles in DOX-induced renal damage in our D-galactose-induced accelerated renal aging model. Consistent with our findings, a study using young healthy rodents also demonstrated oxidative impairment and mitochondrial dysfunction after DOX administration [28,29]. In addition, mitochondrial transplantation has been found to ameliorate DOX-induced mitochondrial and cellular toxicity in renal proximal tubular cells of young healthy rats [30].

A major mechanistic finding in our study is the dysregulation of mitochondrial dynamics following DOX treatment. Increased phosphorylation of DRP1 and downregulation of fusion proteins Mfn1, Mfn2, and OPA1 indicate a shift toward excessive mitochondrial fission. This imbalance promotes mitochondrial fragmentation, oxidative stress, and facilitates the release of apoptogenic factors, contributing to intrinsic apoptosis. The elevation of cleaved caspase-3/procaspase-3 and the Bax/Bcl-2 ratio further confirms the activation of mitochondria-mediated apoptotic pathways. These results are consistent with emerging evidence that mitochondrial dynamics, specifically the balance between fission and fusion, are key determinants of cell survival during renal injury [31,32,33,34].

Mdivi-1 treatment, administered either concurrently with DOX or after the onset of injury, significantly improved mitochondrial morphology and function. It restored mitochondrial membrane potential (MMP), reduced mitochondrial ROS production, and attenuated the dysregulation of key mitochondrial dynamics proteins induced by DOX. Importantly, Mdivi-1 also reduced the expression of apoptotic markers (Bax/Bcl-2 ratio and cleaved caspase-3/procaspase-3 ratio) and PINK1, suggesting attenuation of apoptotic cell death together with reduced mitochondrial stress. Moreover, Mdivi-1 treatment effectively suppressed the DOX-induced upregulation of TNF-α and IL-1β, further supporting its potential to attenuate renal inflammation. Collectively, these renoprotective effects underscore the therapeutic potential of targeting mitochondrial fission as a strategy to mitigate DOX-induced nephrotoxicity in the context of renal aging.

A notable finding of this study is that Mdivi-1 conferred comparable renoprotective effects when administered either concurrently with DOX or after the onset of injury. This observation suggests that excessive mitochondrial fission contributes not only to the initiation of renal injury but also to its progression. From a translational perspective, these findings indicate that therapeutic targeting of mitochondrial fission may remain beneficial even after kidney injury has been established, thereby broadening the potential therapeutic window for mitigating DOX-induced nephrotoxicity in the context of renal aging.

## 5. Limitations and Future Directions

Although our findings support the therapeutic potential of Mdivi-1 in DOX-induced nephrotoxicity, several limitations should be acknowledged. First, although we employed a well-established D-galactose model to simulate renal aging, we did not directly measure senescence markers such as SA-β-gal staining or *p*16^INK4a^ expression due to technical constraints. Nevertheless, this model has been widely validated in previous studies as a reliable surrogate of renal aging in kidney and mitochondrial research [14,16]. Second, although Mdivi-1 is a widely used DRP1 inhibitor, our study did not include an Mdivi-1 standalone control group on either the normal or D-galactose background, meaning potential independent baseline or off-target effects cannot be entirely excluded [35]. Therefore, future studies employing an inhibitor-alone control group or genetic DRP1 knockout or knockdown models would further strengthen the mechanistic evidence. Third, we did not evaluate markers of mitochondrial biogenesis (such as PGC-1α, Nrf1, and TFAM) and the complete downstream mitophagy cascade beyond the upstream sensor PINK1 (such as Parkin, LC3-II, and p62) due to technical constraints. Consequently, distinguishing between compensatory and excessive autophagic pathways remains limited, and these combined markers would provide a more comprehensive understanding of mitochondrial turnover and recovery. Finally, this study used only female rats; therefore, sex-specific differences in mitochondrial regulation may limit the generalizability of the findings. Future studies should address these limitations using both pharmacological and genetic approaches in naturally aged or tumor-bearing models to better reflect clinical scenarios. Moreover, time-course analyses to define the therapeutic window for DRP1 inhibition, together with evaluation of long-term outcomes such as persistent renal dysfunction and progression to chronic kidney disease, warrant further investigation.

## 6. Conclusions

This study demonstrates that doxorubicin induces significant renal injury in aging rats through mechanisms involving oxidative stress, inflammation, mitochondrial dysfunction, and dysregulation of mitochondrial dynamics. Pharmacological inhibition of DRP1-mediated mitochondrial fission with Mdivi-1 effectively improved mitochondrial integrity and function, reduced apoptosis, and ameliorated renal injury. Importantly, the comparable efficacy of Mdivi-1 administered as either a coTx or postTx intervention underscores the translational potential of targeting mitochondrial fission, suggesting that DRP1 inhibition could serve as a preventive strategy during simultaneous chemotherapy or as a rescue therapy for patients who develop subsequent nephrotoxicity. Collectively, these findings identify mitochondrial dynamics as a promising therapeutic target for mitigating DOX-induced nephrotoxicity in the context of renal aging and provide a strong rationale for further preclinical and translational investigation. To further translate these findings into clinical settings, future research should focus on three key areas: genetic intervention strategies (e.g., DRP1 knockdown/knockout) to confirm target specificity, in vitro cellular models to track precise signaling mechanisms, and tumor-bearing animal models to ensure DRP1 inhibition does not interfere with doxorubicin’s anticancer efficacy. Finally, because these outcomes were established specifically in a female, D-galactose-induced accelerated aging model, future studies are required to validate the generalizability of this therapeutic scope in males and naturally aged contexts.

## Data Availability

The original contributions presented in this study are included in the article. Further inquiries can be directed to the corresponding author.

## Supplementary Materials

The following supporting information can be downloaded at: https://www.mdpi.com/article/10.3390/biom16081112/s1. Table S1: The details of antibodies used in the study; Figures S1–S3: The original WB images of Figure 6a–c; Figures S4–S6: The original WB images of Figure 7a–c.
