# Supplementary material for: Inhibition of Mitochondrial Fission by Mdivi-1 Alleviates Doxorubicin-Induced Nephrotoxicity in a Rat Model of D-Galactose-Induced Accelerated Renal Aging
Source: Biomolecules. 2026 Jul 29;16(8):1112. doi: 10.3390/biom16081112 (PMC13511591; doi:10.3390/biom16081112)
Supplement: Supplementary file 1 [file biomolecules-16-01112-s001.zip › Table S1 (details of Ab used in the study.pdf]

Table S1. The details of antibodies used in the study

Antibodies	Dilution	Catalog Number	Provider
OPA1	1:1000	80471	Cell Signaling
DRP1	1:1000	5391	Cell Signaling
phospho-DRP1	1:1000	63940	Cell Signaling
Mfn2	1:1000	9482	Cell Signaling
Bax	1:1000	2772	Cell Signaling
pro caspase-3	1:1000	14220	Cell Signaling
cleaved caspase-3	1:1000	14220	Cell Signaling
β-Actin	1:1000	8457	Cell Signaling
VDAC	1:1000	4661	Cell Signaling
Anti-Rabbit	1:2000	7074	Cell Signaling
PINK1	1:1000	ab326097	Abcam
Mfn1	1:1000	ab221661	Abcam
Bcl-2	1:1000	ab196495	Abcam
