# Supplementary material for: Inhibition of Mitochondrial Fission by Mdivi-1 Alleviates Doxorubicin-Induced Nephrotoxicity in a Rat Model of D-Galactose-Induced Accelerated Renal Aging
Source: Biomolecules. 2026 Jul 29;16(8):1112. doi: 10.3390/biom16081112 (PMC13511591; doi:10.3390/biom16081112)

Figure: S1 The original WB images correspond to Figure 6(a)

pDRP1 (80 kDa)

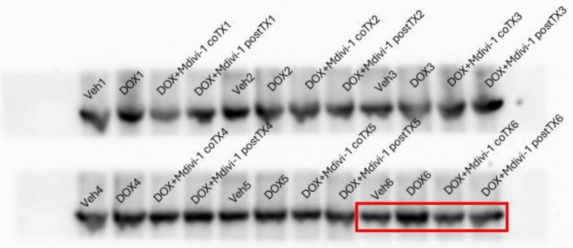

VDAC (32 kDa)

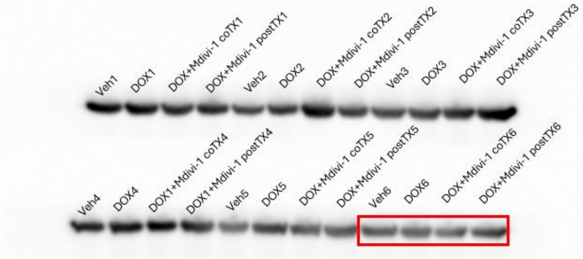

DRP1 (80 kDa)

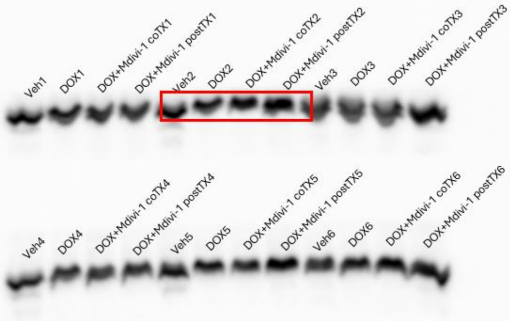

VDAC (32 kDa)

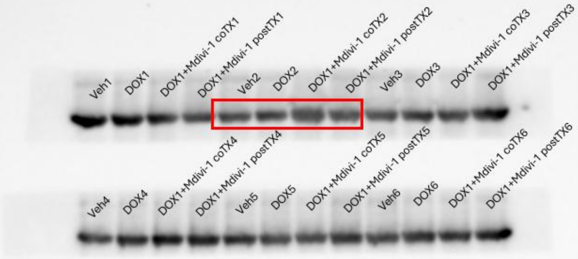

Figure: S2 The original WB images correspond to Figure 6(b)

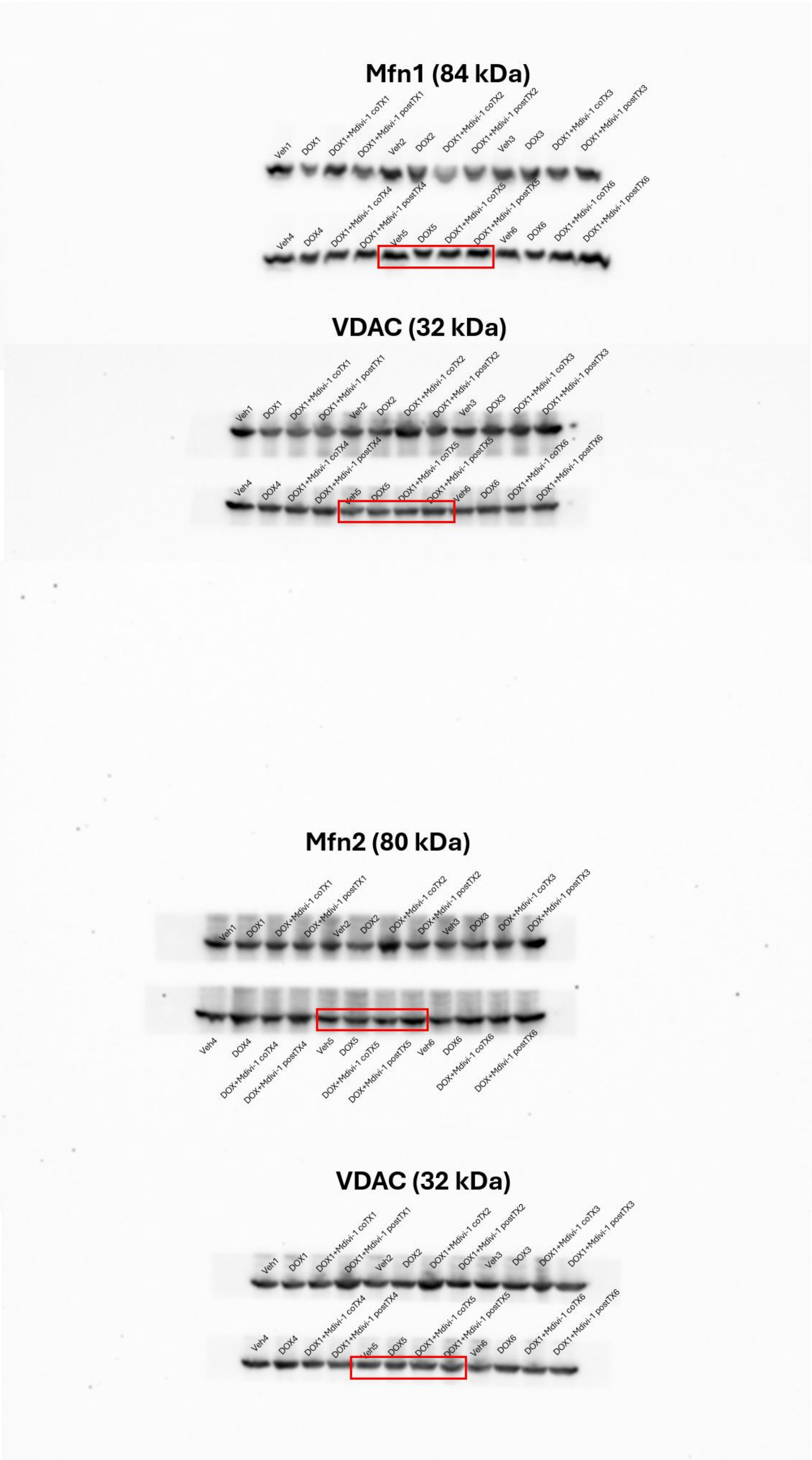

Figure: S3 The original WB images correspond to Figure 6(c)

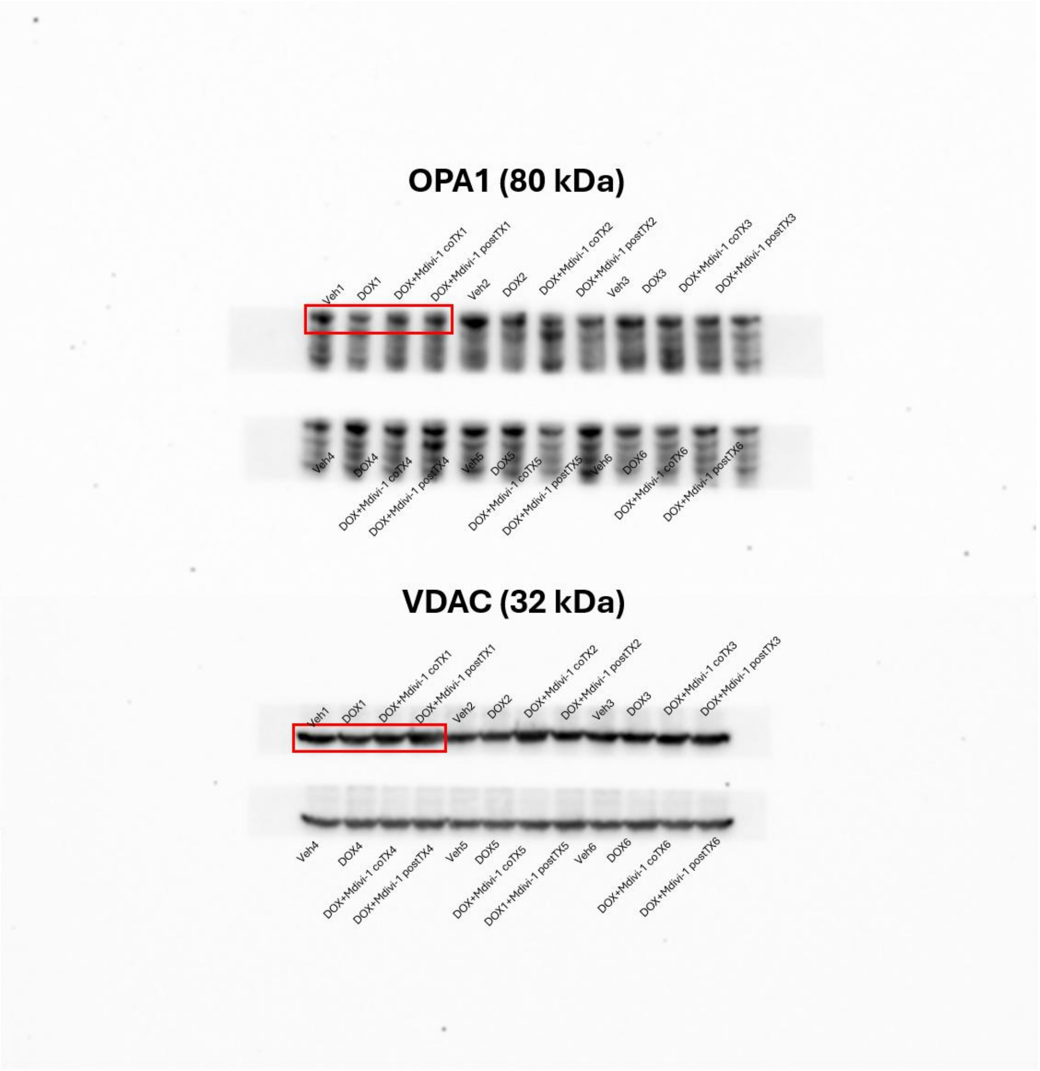

Figure: S4 The original WB images correspond to Figure 7(a)

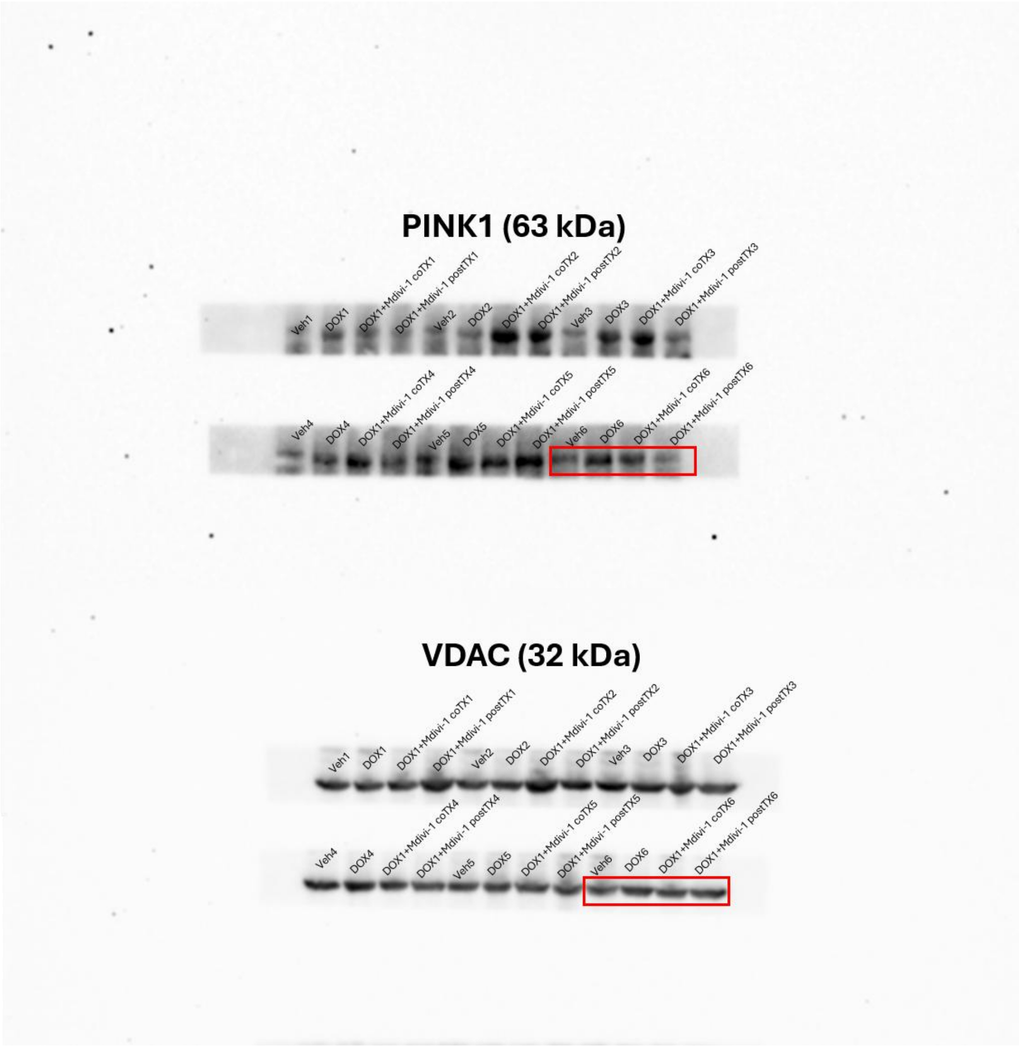

Figure: S5 The original WB images correspond to Figure 7(b)

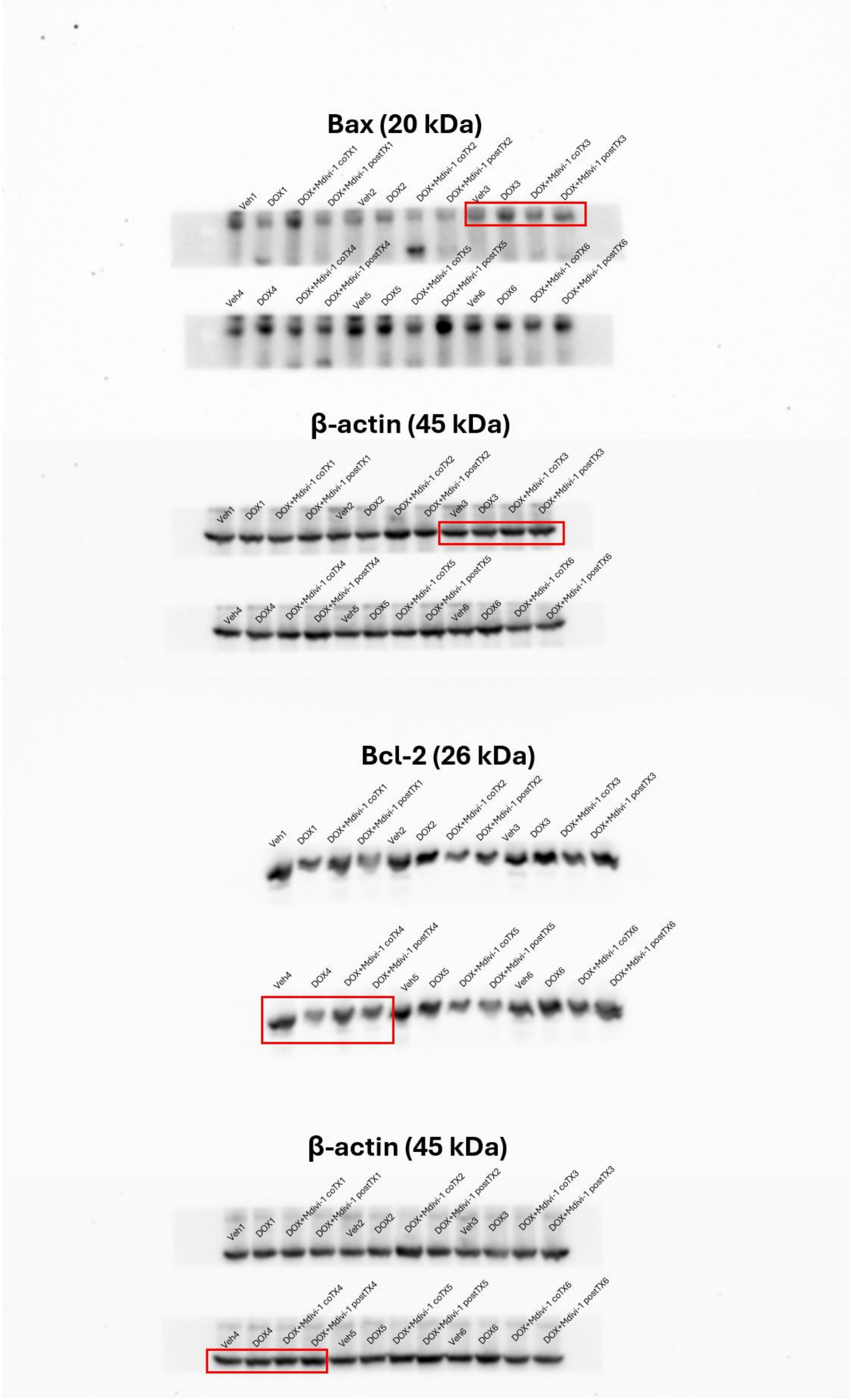

Figure: S6 The original WB images correspond to Figure 7(c)

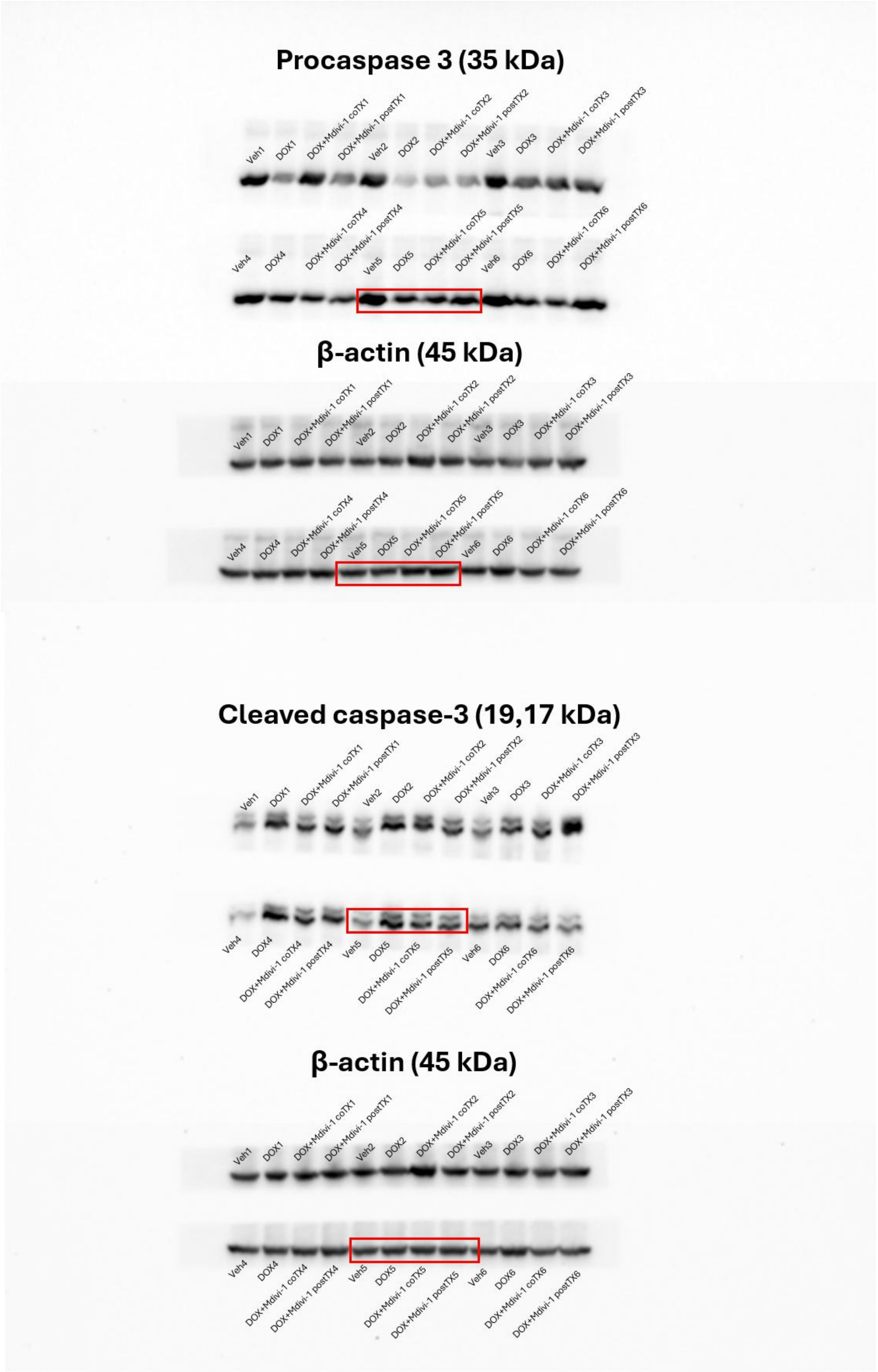

Supplement: Supplementary file 1 [file biomolecules-16-01112-s001.zip › Figure S1-6 original WB images.pdf]
